# Characterizing the Patient Journey in Multiple Myeloma: Qualitative Review

**DOI:** 10.2196/39068

**Published:** 2022-09-22

**Authors:** Matthew Lyall, Rebecca Crawford, Timothy Bell, Carla Mamolo, Alexander Neuhof, Courtney Levy, Anne Heyes

**Affiliations:** 1 RTI Health Solutions Manchester United Kingdom; 2 SpringWorks Therapeutics Stamford, CT United States; 3 Pfizer Inc Groton, CT United States; 4 Pfizer Pharma GmbH Berlin Germany

**Keywords:** multiple myeloma, literature review, patient-centered insights, patient experience, patient perspectives, patient-reported information, social media, YouTube

## Abstract

**Background:**

The patient experience of multiple myeloma (MM) is multifaceted and varies substantially between individuals. Current published information on the patient perspective and treatment of MM is limited, making it difficult to gain insights into patient needs regarding the condition.

**Objective:**

In this review, a combined research method approach (ie, the review of published literature and social media posts) was undertaken to provide insight into patients’ perspectives on the burden and treatment of MM, the impact of the COVID-19 pandemic, and the impact of MM on caregivers of patients with MM.

**Methods:**

Targeted searches of PubMed and PsycINFO were conducted from November 16, 2010, to November 16, 2020; in parallel, patient-reported information derived from social media posts from 6 patient advocacy websites and YouTube were searched. The review of patient advocacy websites and YouTube targeted patient-reported information from patients with a self-reported diagnosis of MM who discussed their experience of MM and its treatments.

**Results:**

A total of 27 articles and 138 posts were included (patient-reported information included data from 76 individuals), and results from both sources showed that patients experienced a variety of symptoms and treatment side effects, including neuropathy, fatigue, nausea, and back pain. These can affect areas of health-related quality of life (HRQOL), including physical functioning; emotional, psychological, and social well-being; the ability to work; and relationships. Patients valued involvement in treatment decision-making, and both the patient-reported information and the literature indicated that efficacy and tolerability strongly influence treatment decision-making. For patients, caregivers, and physicians, the preference for treatments was strongest when associated with increased survival. Caregivers can struggle to balance care responsibilities and jobs, and their HRQOL is affected in several areas, including emotional-, role-, social-, and work-related aspects of life. The COVID-19 pandemic has challenged patients’ ability to manage MM because of limited hospital access and restrictions that negatively affected their lives, psychological well-being, and HRQOL. Unmet patient needs identified in the literature and patient-reported information were for more productive appointments with health care professionals, better-tolerated therapies, and more support for themselves and their caregivers.

**Conclusions:**

The combination of published literature and patient-reported information provides valuable and rich details on patient experiences and perceptions of MM and its treatment. The data highlighted that patients’ HRQOL is impeded not only by the disease but also by treatment-related side effects. Patients in the literature and patient-reported information showed a strong preference for treatments that prolong life, and patients appeared to value participation in treatment decisions. However, there remain unmet needs and areas for further research, including treatment, caregiver burden, and how to conduct appointments with health care professionals. This may help improve the understanding of the journey of patients with MM.

**Plain Language Summary:**

Multiple Myeloma (MM) is the second most common cancer that affects blood cells. In this study, researchers wanted to know patients’ views on the effects of MM and the treatments they received. Researchers also looked at the impact of the COVID-19 pandemic on patients’ treatment and the impact of MM on caregivers. To this end, the researchers reviewed information from 27 published studies and 138 social media posts by 76 patients with MM. Patients commonly reported nerve pain, tiredness, feeling sick, and back pain caused by MM and the treatments they received. The effects of MM and treatments affected patients’ physical function; emotional, psychological, and social well-being; ability to work; and relationships. The researchers found that patients wanted to be involved in decisions related to their treatment. The effectiveness against MM and known negative effects strongly influenced the choice of treatments for patients. Increased survival was the strongest factor in the choice of treatment for patients, caregivers, and doctors. Researchers found that the emotional-, role-, social-, and work-related aspects of caregivers’ lives were affected by caring for patients with MM. The COVID-19 pandemic also affected the ability of patients to manage their MM because of limited hospital access and the effects of restrictions that impacted their lives and psychological well-being. Finally, the researchers identified some areas requiring improvement, including unproductive appointments with health care professionals, the need for treatments with fewer negative effects, and more support for patients with MM and their caregivers. This information may be useful to improve and understand the experience of patients with MM.

## Introduction

### Background

Multiple myeloma (MM) is an incurable systemic hematologic malignancy typically characterized by the neoplastic proliferation of plasma cells and the production of monoclonal immunoglobulins from these cells [1,2]. It accounts for approximately 1% of all cancers and, after lymphoma, is the second most common hematologic malignancy, with an age-standardized incidence of 5 in 100,000 cases in the Western world. Most cases occur in patients aged >65 years and develop from a monoclonal gammopathy of unknown significance, with the risk of progression from monoclonal gammopathy of unknown significance to MM estimated at 1% of cases a year [1,3,4]. MM is a heterogeneous disease that is relapsing-remitting in nature; nearly all patients relapse or become refractory to treatment [1,3]. The overall median survival in patients with MM is >5 years but, because of its unpredictable course of progression, some patients go for extended periods without needing treatment, whereas others experience disease progression and rapid decline in health, often not responding to treatment [1,3,5].

Bone destruction, marrow failure, and complex organ dysfunction are some of the consequences of the characteristic neoplastic proliferation of tumor cells in MM, which can lead to a range of symptoms that are amplified and accelerated during relapses, placing a substantial symptom burden on health-related quality of life (HRQOL) [1,2,6]. Furthermore, nonspecific symptoms are common and may be present for extended periods before diagnosis. These can include impaired renal function, anemia, pain, and weight loss [7]. Thus, patients with MM often require informal care (eg, from partners), which can increase the emotional, social, and work impact on both patient and caregiver [8].

The development of a range of therapies for MM over the past 2 decades has led to an improvement in overall survival [4,7]. However, many therapies are associated with detrimental side effects that can severely affect HRQOL [3,9,10]. Patients are often prescribed disease-modifying therapies such as chemotherapy, immunomodulatory agents, and proteasome inhibitors that can cause side effects such as gastrointestinal symptoms, cognitive effects, and substantial neuropathy [3,10,11]. Analgesics such as steroids and opioids are commonly prescribed for pain caused by disease-modifying therapies or MM itself and are associated with cumulative toxicities that can result in side effects such as pain, fatigue, and sleep disturbances [3,9-11]. Consequently, therapeutic management of MM is challenging and is a significant area to consider when assessing disease burden [1,3,5]. The management and burden of cancer has been further complicated by the COVID-19 pandemic (November 2019-present) because of the increased risk of severe infection and its impact on access to health care and medical services. This may be potentially salient for patients with cancer because of their immunosuppressed status caused by chemotherapy or the disease itself; however, there are limited data available [12,13].

As a result of treatment side effects and the complex nature of MM, the patient experience is multifaceted and varies substantially between patients and at different time points of the disease. Published information on the patient perspective of MM and its treatment is limited, making it difficult to gain insights into patient needs regarding the condition [5]. Patient-reported information provides a valuable source of unsolicited data that could help gain a better understanding of the patient perspective. Social media data have been defined as information reported by patients (or caregivers) outside the formal research context relating to their experience of the disease and its treatment [14]. The US Food and Drug Administration guidance has indicated that social media searches may be useful in complementing literature review findings for insight gained regarding the patient experience of symptoms and disease impact [15].

### Objectives

This study used a combined research method approach (ie, review of published literature and social media posts) to identify information in the patients’ voice on the burden and treatment of MM, the impact of the COVID-19 pandemic, and the impact on caregivers, providing an up-to-date assessment of the burden of MM from the patient perspective.

## Methods

### Targeted Literature Review

A targeted review of the published literature in PubMed (via the National Library of Medicine Gateway) and PsycINFO was conducted from November 16, 2010, to November 16, 2020, using a study-specific search strategy to identify recent information in the patients’ voice on the burden and treatment of MM. The search strategy was limited to the English language and humans and excluded commentaries, letters, and editorials. Titles and abstracts of the identified articles were screened (single screening; 1 reviewer per record), and the most recent articles describing the patient perspective on MM burden, treatment, costs, caregiver burden, and COVID-19 pandemic impact were selected for inclusion. A targeted desktop search of the American Society of Clinical Oncology and the International Society for Pharmacoeconomics and Outcomes Research websites was also conducted to identify relevant data from recent conferences that were not available in PubMed.

### Social Media Review

The targeted literature review was supplemented with a targeted review of social media data to identify patient-reported information on the patient experience of MM. A pragmatic Google search was conducted to identify patient advocacy websites hosting patient-contributed content. The Google advanced search function was used to identify web pages that included “multiple myeloma” in conjunction with the following key search terms: “patient narratives,” “patient stories,” “patient advocacy,” and “patient organization.” The results were then reviewed to identify MM patient organizations and other websites that might contain patient-reported information that described the patient experience of MM and its treatment. Website content was reviewed for relevant patient-reported information; sites presenting irrelevant patient-reported information were not included. Six relevant patient advocacy organizations were identified: CURE, The Patient Story, PeopleBeatingCancer, Myeloma Crowd, Multiple Myeloma Research Foundation, and Patient Power [16-21]. Their websites provide information and support for people affected by cancer, including interviews conducted with patients, caregivers, and patient advocates focused on specific cancers and treatments. All 6 websites included relevant patient-reported information. Only publicly available information was reviewed, and permission was sought from the organizations to use content from their websites for the review. A search of YouTube was also conducted using “multiple myeloma” in conjunction with key search terms (“patient narratives,” “patient stories,” “patient journey,” and “COVID-19”) to identify any further relevant MM-related patient-reported information. YouTube is a global web-based platform where registered users can easily upload and share videos; videos uploaded with “public” privacy settings can be viewed by any internet user. The social media review was conducted during the COVID-19 pandemic (November 2020); thus, it was important that the review was sensitive to the patient lived experience of the pandemic and the potential consequences for their wider HRQOL. The key search terms used to identify patient-reported information within the websites and YouTube are listed in Multimedia Appendix 1.

The review of patient advocacy websites and YouTube targeted patient-reported information from patients with a self-reported diagnosis of MM who discussed their experience of MM and its treatments. Posts were considered eligible for inclusion if they were shared by adults (aged ≥18 years) with a self-reported MM diagnosis, if the adult patient and not a proxy (eg, caregiver, physician, or relative) contributed to the patient-reported information themselves, if the post was in English, and if the content was relevant to patient MM experience and treatment. All video footage and blog posts were manually reviewed to determine eligibility for inclusion in the review. Where available, patient demographic and disease characteristics were extracted manually (annotation-based) from the social media posts. The content of the social media posts was analyzed thematically by independent researchers—one researcher extracted the patient-reported information and used a combined deductive and inductive approach for coding the text; a second researcher reviewed the coded text and discussed any issues with the first researcher (major themes and codes used to analyze the patient-reported information are presented in Multimedia Appendix 2). The results were then summarized based on agreed themes that were derived from the research questions or that emerged from the social media text.

### Ethical Considerations

The RTI International Institutional Review Board determined that this study did not constitute research with human participants (STUDY00021421).

## Results

### Search Findings

The literature search identified 374 articles, of which 27 (7.2%) relevant ones were selected for potential inclusion in the review. Desktop searches of conference websites identified 5 further abstracts from the American Society of Clinical Oncology and the International Society for Pharmacoeconomics and Outcomes Research. The literature identified covered areas of disease overview and burden to the patient, burdensome symptoms, treatment expectations and goals, patient preferences on treatment attributes, cost burden to the patient, impact on caregivers, decision-making (treatment), adherence, and unmet needs.

**Figure 1:**
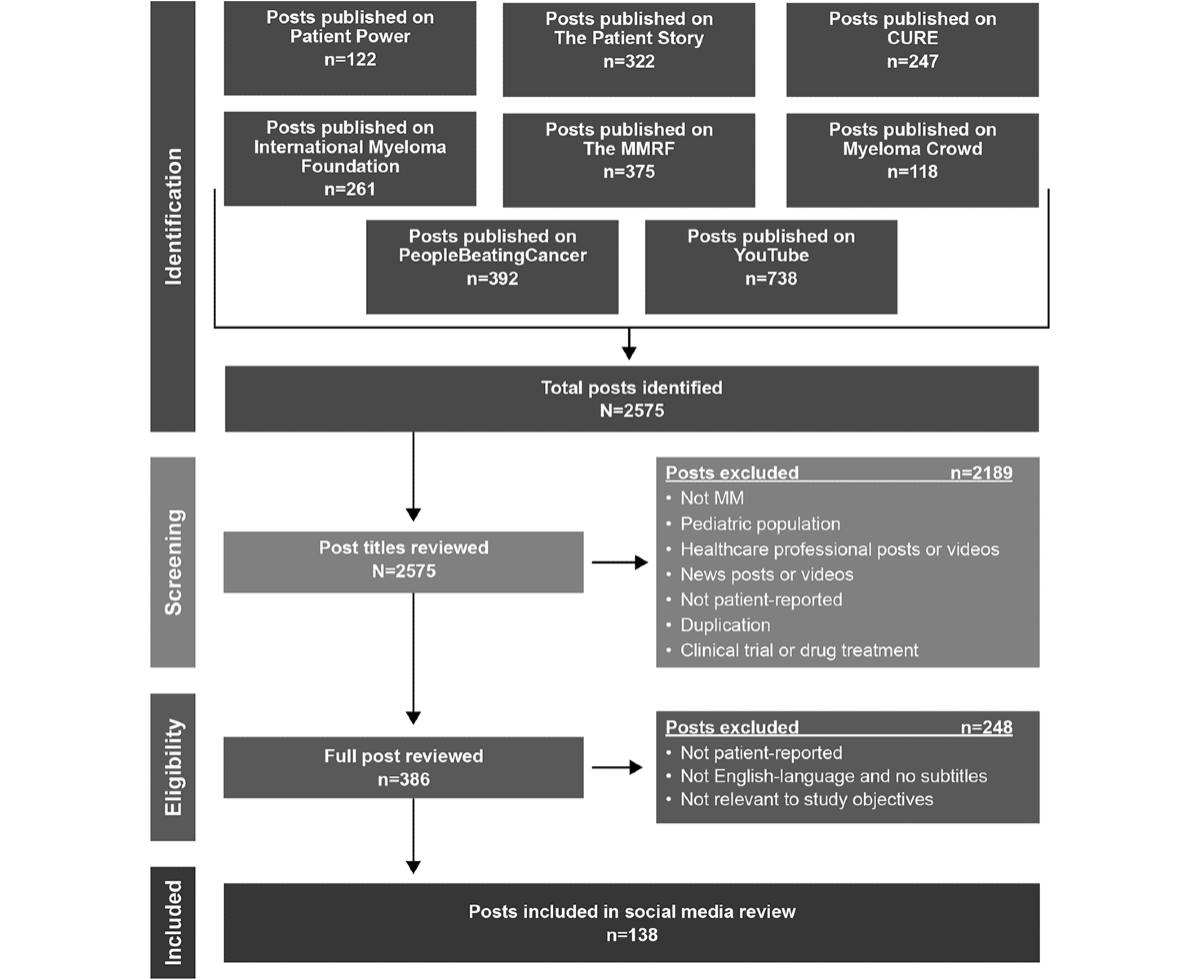
Social media postidentification flowchart. MM: multiple myeloma; MMRF: Multiple Myeloma Research Foundation.

The social media review identified 2575 social media posts, which were evaluated against prespecified review criteria, and 138 (5.36%) posts were identified as relevant for the final review (Figure 1): 79 (57.2%) videos (totaling 10 hours, 19 minutes, and 32 seconds of footage), 58 (42%) blog posts, and 1 (0.7%) podcast. The 138 social media posts included patient-reported information from 76 unique contributors, half of whom (n=38, 50%) were male. Age was available for 24% (18/76) of the contributors and ranged from 36 to 71 years.

### Key Themes

Table 1 shows a summary of the key themes that emerged from the targeted literature review and social media review.

**Table 1:** Summary of key topics and themes that emerged from the targeted literature review and social media review.

Topic	Targeted literature review: key themes	Social media review: key themes
Symptom experience of MM^a^	Pain (back pain and bone pain), fatigue, nausea, and dyspnea	Pain (back pain, *general pain^b^, rib pain, sternum pain, hip pain,* and *knee pain*), *neck pain*, and bone pain; *fractures*; fatigue; *infection; lesions;* and *tumors*
Patient HRQOL^c^	Impact on physical functioning (limitations on physical activity and moving around and increased fatigue and exhaustion) Impact on role functioning (concerns regarding eating and nutrition) Impact on social functioning (disrupted day-to-day life because of exhaustion and hospital visits) Psychological and emotional impact (depression, anxiety, and reduced social satisfaction)	Impact on physical functioning (restricted physical activity or mobility) Impact on daily life (day-to-day activities, hobbies and leisure activities, and rest) Psychological and emotional impact (impact of reaction to diagnosis [devastation and shock], fear of the future, depression, anxiety, denial, frustration, feeling isolated, anger, feeling lost, changed perception of self, and positive emotions [gratitude]) *Impact on relationships (change in relationships with family and friends, impact on relationship with children, impact on relationship with partner, and partner becoming a caregiver)* *Impact on work and finances (inability to work, employment issues, and financial burden)*
MM treatment experience	Type of treatment (opioids, analgesics, chemotherapy, immunomodulatory drugs, proteasome inhibitors, CD38 inhibitors, and steroids) and treatment efficacy (analgesics helped relieve pain but were associated with side effects)	Type of treatment (*general stem cell transplant*, chemotherapy, *radiation therapy, CAR-T^d^ therapy, surgery, and clinical trials*), *treatment efficacy (lack of durable efficacy from treatments and quick efficacy from CAR-T treatment), and treatment administration type (infusions were quick and injections were disliked because of bruising)*
Treatment experience	Treatment impact: Function and mobility Uncertainty Disruption to daily life Psychological and emotional well-being Isolation and negative effect on relationships Financial impact Sleep disturbances Treatment side effects: Neuropathy, tiredness, musculoskeletal pain, fractures, diarrhea, and cognitive impairment Chemotherapy: gastrointestinal and cognitive side effects (chemo fog) Opioids: fatigue, constipation, and dizziness Steroids: pain, fatigue, infections, nausea, and sleep disturbances Treatment could exacerbate MM symptoms (eg, steroids), and there was an overlap between MM symptoms and side effects Treatment with opioids was sometimes stopped or reduced to prevent side effects Experience of treatment side effects can be acute but also chronic	Treatment impact: Treatment burden (travel to appointments, isolation from family and friends, cost of medication, and chemotherapy holiday) *Impact on work (returning to work after treatment and continuing to work while on treatment)* Treatment side effects: Neuropathy, fatigue, nausea, infection, chemotherapy-induced cognitive dysfunction (chemo brain or brain fog), sleep disturbance, chemotherapy-induced cardiomyopathy, secondary cancer, graft vs host disease, bone structural problems, water retention, gastrointestinal symptoms, low testosterone levels, blood clots, hair loss, hallucinations, vomiting, diarrhea, headaches, cytokine release storm, rash, low blood pressure, radiotherapy-induced lumbosacral plexopathy, muscle loss, aches, nosebleeds, anemia, general pain, confusion, and forgetfulness Treatment side effects were long-lasting *Treatment side effects built up over time* *Burden of steroid use:* *Weight gain, sleep disturbances, irritability, acid reflux, increased appetite, fatigue, hyperpigmentation, and anxiety*
Treatment hopes and preferences	Treatment hopes: To increase survival Treatment preferences: Increased survival, reduced side effects (physical and cognitive), lower financial impacts, independence, and convenience (home administration) Caregivers were less cost-sensitive Physicians were concerned about cost and survival	*Treatment hopes:* *Hope to be cured or cancer-free, treatment not being as effective as anticipated, unexpected relapse, and health care professional influence on the patient’s expectations* Treatment preferences: Caution or skepticism regarding stem cell transplant, fewer drugs, chemotherapy over stem cell transplant, clinical trials to obtain the latest drugs, treatment type and burden, and therapies with a history of good outcomes
Treatment decision-making	Patients showed a preference for contributing to treatment decisions Sharing treatment decisions with physicians was preferred by patients who were not treatment-naïve Trust in health care providers was important for decision-making	Patient having choice over treatment, physicians deciding treatment, *pressure from physicians regarding treatment choice, choosing to stop treatment, and delaying treatment because of family events*
Patient experience of MM during the COVID-19 pandemic	Impact on treatment (reduced access to hospitals for administration) Impact on daily life (pandemic restrictions and boredom) Emotional impact (anxiety surrounding hospital visits and feelings of loneliness, stress, and missing family)	Impact on daily life: Following the COVID-19 guidelines, minimizing time spent outside (eg, walks and shopping), missing out on social life and seeing family and friends, avoiding gyms, and limited information and support from the government Emotional impact: Feeling nervous or vulnerable because of MM, feeling safe and confident, worrying about exposure, anxiety, and fear of the immediate future (eg, impact of COVID-19 on cancer) Impact on treatment: Treatment as usual, delayed treatment, changes to telehealth medical appointments, adaptations to health services, cautiousness over immunosuppression preparing the patient for COVID-19, and limited or no guidance on treatment delivery updates
Impact on caregivers	Emotional impact (uncertainty about the future, isolation, stress, and frustration about the disease prognosis and while waiting for test results) Impact on daily life and work life (hospital visits and running the house restrict time for work and life) Financial impact (reduced time to work) Physical impact (tiredness and fatigue) Strain on relationships (hiding feelings, trying to stay positive, and keeping information from the patient)	Strain on caregiver and strengthened relationship

^a^MM: multiple myeloma.

^b^The text in italics indicates themes identified in the social media review that were not identified in the literature review.

^c^HRQOL: health-related quality of life.

^d^CAR-T: chimeric antigen receptor T-cell.

### The Impact of Disease Symptoms

It is well established that MM is associated with burdensome symptoms, and both the literature (5/27, 19% of the articles) and patient-reported information (44/76, 58% of the patients) identified neuropathy, tiredness, nausea, fractures, and back pain as common MM symptoms [3,10,22-24]. A study that investigated HRQOL concepts reported by patients with MM (N=230) using social media listening methods reported that back pain was a prominent symptom experienced early in the disease course; tiredness, nausea, fatigue, and bone pain were generally reported after MM diagnosis; and neuropathy often came after a relapse [22]. These symptoms affected the physical, functional, emotional, and social aspects of patients’ health [23,25]. The patient-reported information (44/76, 58% of the patients) confirmed that symptom burden was an important aspect of the patient experience of living with MM; symptoms were reportedly persistent and had a detrimental impact on patients’ HRQOL:

Pain, from day to day, is always there, at some level or other, for me.

Male patient, age not reported; patient-reported information

I have fatigue, and people ask me, “Well, how are you able to go out and walk 3, 5 miles, ride your bike, go to the gym?” et cetera. Um, I really push myself, and then I get home, and I collapse.

Female patient, age not reported; patient-reported information

Compared with the general population, patients with MM reported a reduced HRQOL [23-25]. A prospective study of patients with MM (n=156) ≤10 years after diagnosis reported that patients experienced substantial symptom burden and poor HRQOL regardless of the time since diagnosis [25]. Both short-term (<5 years) and long-term (≥5 years) survivors had statistically significantly and clinically relevant worse HRQOL scores when compared with a normative population (n=500), and clinically important inferior scores (as measured by the European Organization for Research and Treatment of Cancer Core Quality of Life questionnaire and Multiple Myeloma Module) were greatest for quality of life (42%), physical functioning (42%), role functioning (41%), dyspnea (41%), and social functioning (38%) [25].

Symptoms of MM substantially affect physical function; this limits daily activities and causes psychological distress [24]. Zaleta et al [23] investigated 283 patients with MM using the CancerSupportSource 25-item distress screening tool, which examines physical, social, emotional, and practical concerns. Strongly patient-endorsed concerns regarding MM included eating and nutrition (61%), exercising and being physically active (59%), moving around (56%), and feeling too tired to do things that patients needed or wanted to do (55%). Impaired physical functioning and fatigue were reported by 38% and 33% of patients, respectively. Only 27% of patients reported that they believed that they had control over the course of their MM. Patients also reported impaired psychosocial well-being in areas relating to depression (17%), anxiety (20%), and social satisfaction (29%) [23]. Similar results regarding the debilitating impact of MM on patients’ HRQOL emerged from the patient-reported information; Table 2 shows some of the areas of HRQOL affected by MM as well as themes and patient quotes associated with these areas. Over 55% of the patient contributors (44/76, 58%) discussed the impact of MM on various areas of their HRQOL, including physical functioning, emotional and psychological well-being, ability to work, and relationships.

**Table 2:** Key areas of health-related quality of life (HRQOL) reported by patients with multiple myeloma (MM; source: social media review; N=76).

Area of HRQOL affected	Patients, n (%)	Example of areas of life affected	Quotes from patients with MM from social media
Physical functioning	11 (15)	Restricted physical activity and mobility Walking Stairs Running and lifting and carrying	“I can’t even...sit down or stand up from my laying position. I can only lay down on the bed with limited movement.” (Male patient, age NR^a^)
Daily activities	9 (12)	Hobbies and leisure Sports and fitness Rest New “norm”	“I have to limit myself now. That can be a struggle...I don’t like not being able to do some of the things I used to be able to do.” (Female patient, age NR)
Work finances	8 (11)	Inability to work Employment issues Financial burden	“I had to give up the dream of both starting a health spa...Instead, just surviving multiple myeloma became my full-time job.” (Female patient, aged 52 years)
Relationships	13 (17)	Change in relationships Change in roles Lack of understanding Loss of friends	“It put a lot of stress and strain on our relationship...He [partner] became more of a caregiver while I became a patient.” (Female patient, age NR)
Psychological and emotional impact	26 (34)	Reaction to diagnosis (devastation and shock) Fear of the future Uncertainty Change in self Mood	“There is a really important psychological aspect to it...If you’re feeling down, miserable...you notice your pain a lot more. There’s no doubt I do.” (Male patient, age NR)

^a^NR: not reported.

### The Impact of Treatment

The prognosis of MM has greatly improved in recent years as a result of the changing myeloma treatment landscape, which has seen the development of a range of treatment options. However, according to the published literature, patients on these treatments experience unpleasant side effects or symptoms that they attribute to their medication and that result in negative impacts on patient HRQOL [3,9,10,22]. Results from a study on patient-reported disease- and treatment-related symptoms—which extracted data from a patient-powered research network—noted that neuropathy was the symptom most frequently reported by patients with MM and that patients specifically discussed neuropathy as a consequence of treatment [22].

In total, 11% (3/27) of the published studies described that patients receiving disease-modifying therapy (eg, chemotherapy) experienced physical effects, including severe tiredness, musculoskeletal pain and fractures, and neuropathy that affected overall function and mobility [3,10,26]. Patients also reported gastrointestinal side effects associated with undergoing chemotherapy (including bendamustine, cisplatin, cyclophosphamide, doxorubicin, etoposide, and melphalan). Patients who experienced gastrointestinal side effects were particularly cognizant of their food choices to minimize or avoid the likelihood of experiencing diarrhea, constipation, and nausea. Cognitive side effects, such as “chemo fog,” losing their “train of thought,” and struggling to retrieve information, were also prominent features of treatment experience with chemotherapy [3,26]. In the patient-reported information, a range of treatments were discussed, including chemotherapy (30/76, 39%), general stem cell transplant (26/76, 34%), radiation therapy (3/76, 4%), chimeric antigen receptor T-cell therapy (2/76, 3%), surgery (2/76, 3%), and treatments in clinical trials (9/76, 12%). Treatment-associated symptoms and the resultant detriments to patients’ health were discussed by 29% (22/76) of the patient contributors. The most prominently discussed examples of treatment-associated symptoms were neuropathy (3/76, 4%), fatigue (3/76, 4%), nausea (2/76, 3%), infection (2/76, 3%), chemotherapy-induced cognitive dysfunction (2/76, 3%), sleep disturbance (2/76, 3%), and chemotherapy-induced cardiomyopathy (2/76, 3%).

Since the literature review was conducted, numerous articles have been published discussing patient experience with disease-modifying MM treatment; in these articles, the negative effects attributed to treatment are still being reported [27,28]. In an exploratory investigation into concepts that influenced treatment choices for patients with MM and that analyzed patients (N=30) receiving proteasome inhibitors (66.7%), immunomodulatory drugs (56.7%), chemotherapy (30%; bendamustine, cisplatin, cyclophosphamide, doxorubicin, etoposide, and melphalan), steroids (70%), and CD38 inhibitors (16.7%), peripheral neuropathy (90%) was the most reported symptom attributed to treatment, followed by diarrhea (83%) and cognitive impairment (83%) [28]. Patients also stated that there was an overlap between symptoms of MM and potential treatment side effects, meaning that they were sometimes unsure if symptoms were caused by treatment or MM [28]. A qualitative study by Nathwani et al [29] investigated adult patients with relapsed and refractory MM (RRMM) who had a life expectancy of ≥3 months and had at least one treatment regimen with a proteasome inhibitor and immunomodulator or a steroid in addition to either a CD38 monoclonal antibody or an alkylating agent. At the time of enrollment, patients (N=22) were treated with regimens containing dexamethasone (59.1%), daratumumab (36.4%), carfilzomib (27.3%), and lenalidomide (18.2%). No adverse symptoms of treatment were reported by 27.3% of patients, but back pain and fatigue attributed to treatment were each reported by 40.9% of patients. Treatment-induced physical function limitations (86.4%), emotional impacts (77.3%), MM-related activity limitations (72.7%), and sleep disturbances (63.6%) were reported by most patients [29].

Analgesics are often prescribed for the relief of bone pain owing to MM or pain caused by chemotherapy. However, in both the literature and patient-reported information (7/76, 9% of the patients), patients who had been prescribed opioids reported that they experienced fatigue, constipation, dizziness, and drowsiness, which they associated with their treatment. These treatment-associated symptoms were considered particularly burdensome and affected HRQOL [3,9,10].

The published literature and patient-reported information also identified a range of negative effects that patients associated with the use of steroids. Symptoms such as pain, fatigue, infection, nausea, and sleep and mood disturbances were associated with steroid therapy by patients, particularly those who received dexamethasone [3,9,10]. For some patients, steroid treatment was associated with the exacerbation of symptoms rather than the intended outcome of providing relief [9,22]:

Dexamethasone is a steroid and I hated it. It had the opposite effect on me that it should have. It made me exhausted instead of wired. It also made me very puffy, and I had some hyperpigmentation.

Female patient, age not reported; patient-reported information

Patients who spent time in the hospital with symptomatic MM (N=21) and had received pain medication were assessed in a study that used semistructured interviews conducted by clinicians [9]. A total of 81% of patients received opioids, 76% took paracetamol, 48% had fentanyl patches, and 33% took oxycodone. Although these therapies relieved patients’ pain, patients experienced side effects that included constipation (48%), dizziness (38%), and tiredness and fatigue (38%); almost all treatment-related side effects were rated as severe or moderate. The interviews consisted of questions on pain medications and MM symptoms, and HRQOL was also assessed using items 29 and 30 from the European Organization for Research and Treatment of Cancer Core Quality of Life questionnaire. The pain medication questions focused on side effects directly attributable to patients’ analgesic medications. Fentanyl patches were reported to be responsible for the greatest proportion of side effects, followed by codeine, morphine, and oxycodone. A total of 48% of patients reported that they either ceased or reduced the dose of pain medication at some point during their illness owing to treatment side effects; this was most often reported for codeine [9].

The patient-reported information (22/76, 29% of the patients) indicated that the negative effects attributed to treatments varied in intensity, were long-lasting, and could build up over time:

My feet are continually numb on the bottom...I mean, it’s just—there’s little things that drive you nuts, and you can manage to a point, but that’s about as far as it goes.

Male patient, age not reported; patient-reported information

I live with 5 serious...long-term and late-stage side effects.

Male patient, aged 60 years; patient-reported information

The varying intensity and impact of treatment side effects add uncertainty to patients’ lives [3,10]. Maher and de Vries [10] reported that treatment side effects commonly included infection ranging in intensity from the acute setting (eg, a Hickman line infection) to living with chronic neuropathy because of infections. In addition, treatment-induced fatigue disrupted patients’ day-to-day lives; patients described the fatigue they experienced as “diabolical,” “sheer exhaustion,” and feeling “desperately tired,” or noted that they were bed-ridden as treatment had the tendency to “take your legs out.” These side effects can result in hospital visits or admissions, disruption of daily routines, and impaired well-being [10].

The patient-reported information (14/76, 18% of the patients) included social media posts that discussed the range of limitations and day-to-day life burdens resulting from treatment, including loss of independence (1/76, 1%), diminished psychological well-being (4/76, 5%), disruption because of medical appointments (1/76, 1%), isolation from family and friends (2/76, 3%), and the cost of medication (2/76, 3%). Some patients (2/76, 3%) reported being able to continue working during treatment, whereas others (5/76, 7%) were able to return only after treatment. Patients described taking “treatment breaks” to be free from the negative symptoms associated with treatment so that they could participate in important family activities and life events. The variability in the impact of treatment means that patients are unable to plan for the future and are constantly preoccupied with the threat of physical deterioration [10]. Textbox 1 presents supportive patient quotes from the patient-reported information that illustrate the effect of treatments on patients’ day-to-day lives.

**The effect of multiple myeloma treatment on the day-to-day lives of patients (source: social media review; N=14).**

**Patient-reported treatment effect**

- “And then we started with a treatment protocol. Suddenly, your independence is taken away from you. Your entire life is taken away.” [Female patient, aged 57 years]
- “Now I’ve had this window where I haven’t had treatment, I realize how much different I feel by it not weighing you down all the time, and frustrating you that you can’t do what you want to do.” [Female patient, age not reported]
- “During nontransplant times of my life in the past year, or couple months, where I’ve still been receiving treatments, but they were treatments where I was still able to work, and I was very grateful for that.” [Male patient, age not reported]
- “The main reason why I want to take a break [from lenalidomide treatment] is, next month, my son is getting married, and I’m really hoping that this break will help simmer down my stomach, because I certainly don’t want to be sick at my son’s wedding.” [Female patient, age not reported]

Patients’ treatment experience can also be influenced by factors such as efficacy and formulation. Of the 76 patients who contributed to the patient-reported information, 41 (54%) discussed treatment experiences—treatment effectiveness, impact on health, and treatment administration were key factors of importance:

The doctors that I saw thought that the first transplant would be the best route to go at the time...but unfortunately, 2 months later, the cancer returned.

Male patient, age not reported; patient-reported information

I had come to realize that although chemo had kept me alive for 5 years, it was also slowly destroying my body.

Female patient, aged 51 years; patient-reported information

### Patients’ Treatment Hopes and Preferences

The complex nature of MM treatment can mean that a range of factors affect patients’ treatment preferences, including history of efficacy and safety, formulation, and novelty of therapy. However, both the patient-reported information and the published literature asserted that increased life expectancy and tolerability are the most important factors from the patient perspective [3]. Treatment preferences discussed in the patient-reported information were influenced by existing treatment success, the opportunity to be on fewer drugs, previous treatment experience, the type of treatment, the mode of administration, the impact on patients’ lives and HRQOL, and the opportunity to experience novel treatments:

I would be more willing to trust something that had a long-term track record of success than something new that we really just don’t know that much about.

Male patient, aged 71 years; patient-reported information

One of [the] things that I was considering back then was how the treatment was given. And one of the treatments that I chose was an oral treatment, because that allowed me to continue to be employed.

Female patient, age not reported; patient-reported information

The literature (4/27, 15% of the articles) reinforced increased survival as the highest priority for treatment [3,26,30,31]. For instance, in 4% (1/27) of the studies, increased survival was rated by patients with newly diagnosed MM or RRMM (N=30) as their top treatment feature [26]. Other important features reported in the published literature included physical side effects, cognitive side effects, financial impacts, and independence [3,26,31]. These additional features were considered by long-term survivors of RRMM as a priority as high as life expectancy [3,26,31]. Neuropathy and cognitive side effects were major concerns for most patients (92% and 94%, respectively) and, thus, were considered important in treatment decision-making [3,26,31]. However, most patients were willing to tolerate some side effects and risks in exchange for treatment benefits, which further emphasized increased life expectancy as an important treatment preference for patients [26].

Treatment preferences can vary among patients with MM, their physicians, and caregivers, as demonstrated in a study by Fifer et al [30]. Caregivers were less cost-sensitive and more concerned with HRQOL than patients, and physicians were generally the most concerned with overall survival and cost. However, all groups valued overall survival as the most important feature of treatment [30].

Patients in the patient-reported information expressed high expectations for treatment outcomes, including the desire for a cure:

There is a chance for a cure, but I’m looking for a long remission, drug free.

Female patient, aged 48 years; patient-reported information

However, patient expectations regarding high treatment effectiveness were not always met because of unexpected relapses and short remission periods. Consequently, patients were often disappointed and upset following ineffective treatment:

I’m really bummed out, ’cause 16 months, I really had thought I was gonna get it down low into a...partial response, and I’m not having that. So it is upsetting to me.

Female patient, age not reported; patient-reported information

The attitude of health care professionals also played a role in moderating patients’ high expectations for treatment:

All of the nurses were really negative; the rounding team that would come around every day...they were kind of lowering my expectations. And I found myself...starting to get a little bit bummed out.

Male patient, aged 71 years; patient-reported information

Patient preference and treatment satisfaction can also be influenced by convenience; improved treatment convenience has been shown to be related to preference [26,32]. A study of patients with RRMM (N=160) found that orally administered treatment predicted satisfaction with treatment convenience as patients treated with an all-oral regimen reported statistically significantly higher scores on a convenience scale than patients who received at least one injectable agent (*P*<.001) [32]. Patients also preferred home over hospital administration as it led to improvements in HRQOL, well-being, and activities of daily living because of reduced hospital travel and waiting times [33]. A small study (N=28) of patients treated with subcutaneous or intravenous bortezomib reported that patients may prefer subcutaneous over intravenous administration as the former was reported to be faster and associated with less neuropathy and fewer general side effects. However, no details on this were identified in the patient-reported information, and further research is needed to confirm this finding [34].

### Cost to Patients

MM has a multifaceted economic burden, and many patients have some unmet financial needs because of treatment copayments (in some countries) and travel costs, which are often highlighted as a burden. In some European countries and the United States, the treatments received by patients for MM and other comorbidities have a substantial impact on costs, which can often be greater than the patients’ ability to pay. The often-unmet financial needs of patients with MM can moderate the relationship between psychological morbidity and HRQOL [32,35,36]. In the United States, a study of 160 patients with RRMM found that treatment copays and the costs associated with visits to the clinic contributed the greatest burden to overall costs [32]. A Portuguese cross-sectional study (N=124) found that 91.9% of previously treated patients with MM reported an unmet financial need, and when financial needs were higher, there was a negative relationship between psychological morbidity and HRQOL [35]. In Finland, an observational study assessed MM-related health care resource use and costs in patients with “active” MM (N=97) treated between 2009 and 2016 [36]. An average travel distance of 35.4 km (approximately 22 miles) was reported for health care visits, which placed a substantial financial burden on patients as the mean per-patient travel costs per 28 days ranged from €75.13 (US $76.42) to €447.99 (US $455.68) [36]. No patient-reported information on the cost of MM to the patient was discussed.

### Treatment Decision-making

Patients with MM generally prefer to participate in the treatment decision-making process; evidence from the published literature and patient-reported information suggests that the extent of information available regarding therapy choices and patient confidence in their treating physician are important [37]. Patients were reported to desire a degree of control over their treatment, with a study finding that almost all patients with MM (97%) regarded “involving patients in therapeutic decisions” as important [11]. This was further supported by a study of older patients (aged ≥60 years) with newly diagnosed symptomatic MM (N=20), which found that 95% of patients preferred partial or total control of treatment decisions, 55% preferred sharing control with a physician, and 40% preferred making decisions after seriously considering physician opinions [37].

The patient-reported information supports the perception that patients prefer to participate in treatment decision-making; 25% (19/76) of patients commented on factors related to treatment decisions. Patients expressed a desire to influence treatment decisions but perceived that their views were not always considered. The degree to which patients were able to assert any influence or direct their treatment paths was dependent on external factors, including their own health (5/76, 7% of the patients), relationship with the physician (3/76, 4% of the patients), and available treatment options (5/76, 7% of the patients). Some patients (8/76, 11%) discussed different treatment options with their physicians, whereas 3% (2/76) of the patients reportedly felt pressured to agree to specific treatments. Furthermore, patients reported having to become advocates for themselves in their treatment choices, particularly when they decided to stop or put treatment on hold to participate in family life events:

My oncologist gave me 8 different options...We went through the list, the pros and cons of each of those 8 options...It was very important to me to sort of understand what his thinking was and why he liked this option versus that option and so forth.

Male patient, age not reported; patient-reported information

A study provided some data to suggest that patients who had previously received treatment for MM showed more of a preference for engaging in increased shared treatment decision-making than treatment-naïve patients [38]. The study used semistructured interviews with patients with MM who had a mean age of 64 years (42% male) and a mean time of 58 months since diagnosis. There were two groups included: (1) patients who had received first-line therapy (n=11) or were in the early relapse phase and (2) patients who had received ≥1 previous lines of therapy (n=10) [38]. As with the patient-reported information, the study reported that trust in one’s health care provider was a notable influence on treatment choice for patients on all lines of therapy. However, the first-line group was generally more willing to follow health care provider decisions, whereas the ≥1 previous lines of therapy group considered other sources of information and preferred shared decision-making. Health care professionals discussed treatment factors (eg, efficacy and tolerability) in more general terms with the first-line group but provided more detail to the ≥1 previous lines of therapy group. Although effectiveness and side effects were the greatest influences on patients’ treatment preferences, the ≥1 previous lines of therapy group was less concerned with side effects.

### Impact of COVID-19

The COVID-19 pandemic has challenged patients’ ability to manage their MM by further disrupting their lives, psychological well-being, and HRQOL. The literature review was conducted from 2010 to November 16, 2020; at the time of the review, no articles relating to the impact of COVID-19 on patients with MM were identified. However, the patient-reported information identified 12% (9/76) of the patients who discussed the impact of COVID-19, specifically the fact that the virus exacerbated the psychological impact of MM. Patients expressed concern and anxiety because of their increased vulnerability to infection and, consequently, took additional precautions to limit physical contact with other people:

I am hypogammaglobulinemic, as many myeloma patients are. It means that I have virtually no immune system with which to fight any infection, let alone COVID-19.

Male patient, age not reported; patient-reported information

Restrictions and a medical focus on COVID-19 also impeded patient treatment because of significant disruptions and delays in medical appointments. Patients in the patient-reported information reported changes to telemedicine appointments, limited or no guidance on treatment delivery updates, and delays to transplantations and suspension of clinical trials of novel MM treatments as effects of COVID-19. However, previous experiences of patients with MM with treatment-related immunosuppression helped with the adjustment to the pandemic-specific social restrictions:

Definitely during this current time [having MM has] made things more difficult. I was getting ready to sort of...take back my life in January, but I was having some side effects from the maintenance medication, so it did get pushed back a bit, and then everything closed down. And so I’m still waiting, but I’m used to it now.

Female patient, aged 35 years; patient-reported information

It is important to note that, since the literature review was conducted, there has been an increase in the number of articles published on the impact of COVID-19 on patients’ disease and treatment experiences for a range of diseases [39-41]. Myeloma Patients Europe published a report in June 2021 on the impact of the COVID-19 pandemic on the health care and lives of people with myeloma and amyloid light-chain amyloidosis and their caregivers. The report identified that living with myeloma in Europe during the pandemic was associated with a number of challenges; approximately 60% of people reported that their treatment was negatively affected during the pandemic. This was particularly true for patients who received their medications in hospitals but less so for those taking oral medications at home. The impact of the COVID-19 pandemic varied in different countries. For example, patients in Belgium stated that hospital services continued as normal, but patients in Romania, Poland, and Scotland reported challenges associated with scheduling appointments and travel restrictions as well as limited hospital access. Some patients also reported that they did not want to visit the hospital because of the risk associated with contracting COVID-19 and that this was an area of stress and anxiety for them. Pandemic restrictions had a substantial impact—a total of 67% of patients and caregivers stated that COVID-19 restrictions negatively affected them. Patients described how social distancing during the pandemic affected their emotional well-being, including feelings of loneliness, anxiety, stress, boredom, and missing friends and family [42].

### Impact on Caregivers

Caregivers of patients with MM can experience a substantial impact on their HRQOL as they often neglect their own needs to provide physical and emotional support, which can significantly affect emotional-, role-, social-, and work-related areas of life [8]. In a study of 20 patients with MM and their 16 informal caregivers (mostly spouses), both groups described MM as a “time bomb” because of significant fears and uncertainty about the future [8]. Caregivers reported that they had to stay positive for patients and that there was sometimes a lack of communication between both parties, which led to feelings of isolation and increased the emotional burden. Both groups kept stressful situations regarding MM secret with the aim of protecting the other person, which could stress and strain relationships. The themes and categories contributing to caregiver burden and unmet needs identified in the published literature are reported in Textbox 2 [8].

**Areas of burden and unmet need related to caring for a patient with multiple myeloma for informal caregivers (source: Molassiotis et al [8]).**

**Practicalities of managing a family member with myeloma and the associated burden for caregivers**

- Caregivers experience fear, uncertainty, and frustration surrounding the prognosis of their relative’s myeloma, which was associated with a substantial emotional burden.
- Waiting for results from tests and visits to the hospital can add further levels of emotional burden because of stress, nerves, and fear of a sudden decline in the health of their partner or family member.
- Caregivers reported hiding or filtering information from the patient when communicating results about the seriousness of the myeloma.
- Caregivers reported not dwelling on themselves or their own feelings and “putting on a brave face” to stay positive for their partner or other family member.
- The practicalities of myeloma (eg, hospital visits and running the house) restricted daily life and work life, which was associated with a financial and physical burden for the caregivers.
- Caregivers reported feeling like they had a duty to provide care on their own with no outside help.

**Areas of unmet need**

- Caregivers reported having an unmet need for specific information and communication surrounding the disease and how to properly care for a patient with myeloma.
- There was an unmet need for people or organizations to turn to with problems or questions or for extra support, with caregivers having to rely on family for extra support.
- Caregivers reported an unmet need for someone to talk to for updates on their family member’s condition as physicians could be too technical and more interested in the disease than in how the patient was.

Caregivers often assist in managing complex treatment regimens and monitoring side effects, which can cause a range of emotions and anxiety as well as difficulties in balancing care responsibilities and work [43]. In interviews with caregivers of outpatients with MM in Spain (N=12), the following 4 main themes emerged relating to caregiver burden: adapting to a new life because of MM, commitment to the patient, emotional impact, and experiences related to the care and support received [44]. Only 3% (2/76) of the patients who contributed to the patient-reported information noted that partners often took on the caregiver role, which could both positively and negatively affect relationships. Patients were also cognizant of the ongoing stress that their condition put on their partners or caregivers, which placed additional stress on the patient:

Unfortunately, he became more of a caregiver while I became a patient. I didn’t like that position. I think it brought out a lot of insecurities in me—especially being in a newer marriage. It also has made us stronger throughout the process because we’ve had to get through us [sic]. We’ve been able to turn to each other and rely on each other. I trust him more. It’s made our connection more solid.

Female patient, age not reported; patient-reported information

My wife bore the brunt of it (diagnosis), and it was so hard on her. I think it was surreal for her. She sold the house, we moved, she was still working, she was traveling to [place name redacted] to see me, trying to take care of our daughter who still lived at home, and so much more. A couple of years ago, I looked at her and said, “I’m okay. You need to refill your tank now. You can’t make it 1 more second.”

Male patient, age not reported; patient-reported information

## Discussion

### Principal Findings

This review of published literature and social media data provides a unique and valuable combination of information on patient experiences and perceptions of MM and its treatment. A wide range of factors that influence patients’ experiences were identified, with the literature and patient-reported information aligning on many aspects. Across the literature and patient-reported information, patients were reported to experience a range of MM symptoms and negative effects from treatment, including neuropathy, fatigue, nausea, and back pain. Symptoms have potentially detrimental effects on HRQOL, and evidence suggested that not only are treatment side effects substantial and long-lasting, but they can also exacerbate symptoms of MM and lead to patients stopping treatment [9,22] (patient-reported information: 22/76, 29% of patients). Both the literature and patient-reported information reported that symptoms and treatment side effects affect areas of HRQOL, including physical functioning; emotional, psychological, and social well-being; the ability to work; and relationships [9]. Furthermore, patients reported economic impacts, and almost all patients reported some form of unmet financial need [32,35,36].

A number of influences can affect MM treatment preference, but both the patient-reported information and literature assert that treatment efficacy and tolerability have a strong influence on treatment decision-making from the patient perspective [3,26,31]. However, increased life expectancy appears to be valued above all else among patients, with evidence from the published literature adding that caregivers and physicians shared this view. Even severe side effects were acceptable in exchange for some treatment efficacy. Owing to heterogeneity in the data, limited sample sizes, and a lack of detail on patient characteristics, conclusions regarding treatment preferences in newly diagnosed MM versus RRMM are limited [26,30,38]. The published literature also suggests that treatment formulation may influence treatment preference, with patients preferring therapy convenience, such as home treatment with reduced travel and treatment duration [26,34]. A desire to be involved in treatment decisions was a strong theme that emerged from both the published literature and patient-reported information [11,37]. Patients valued input on treatment decision-making with physicians as they expressed a desire to share control; however, patients often felt as if their views were not considered [11,37,38]. It may be of value to further explore key themes that emerged from the patient-reported information regarding treatment decisions to investigate if there are additional factors that influence the level to which patients desire to be involved in treatment decisions (eg, whether the line of therapy a patient is on influences their treatment decisions) [38].

Caregivers of patients with MM experience a substantial burden and can struggle to balance care responsibilities and jobs. The published literature and patient-reported information reported that caregiving responsibilities can strain relationships with the patient, but the patient-reported information also found that relationships could be made stronger [8,43-45]. Social media provide patients with platforms to express their opinions and share their experiences in an unstructured way, which can help capture emotions and opinions that may not be captured by traditional research methods. Social media data also allow instant access to unfiltered patient narratives, providing timely information on changes to patients’ disease experiences or challenges patients encounter resulting from external events. This was notable in relation to exploring the impact of the COVID-19 pandemic on patients with MM. The patient-reported information included discussion from 12% (9/76) of the patients regarding the impact of COVID-19, whereas, because of the timing of the targeted literature review, only articles that were published before the COVID-19 pandemic were included in the review. This demonstrates the value of patient-reported information in terms of capturing important aspects of the patient experience as they happen in real time. However, the patient-reported information captured was still limited; therefore, future published studies would help confirm the findings of this study. Following the completion of the literature and social media reviews, data were published on the impact of the COVID-19 pandemic on MM; key issues highlighted included limited access to treatment and hospital services as well as the negative effects owing to COVID-19 restrictions, such as isolation and anxiety [42]. The literature review also provided limited evidence on treatment adherence from the patient perspective; some information indicated that adherence to immunomodulatory drugs is good among patients with MM, but published real-world data and patient-reported information were not available [46].

Unmet needs for patients with MM identified in the published literature and patient-reported information included a lack of productive time with health care professionals, with patients stating that earlier access to results and more time for appointments could help reduce anxiety and maximize discussion time [47]. Better-tolerated therapies, particularly with respect to reduced fatigue and peripheral neuropathy, are needed, and gaps in service provision for patients were identified, such as providing support for patients in coming to terms with the chronic nature of MM and providing advice and reassurance for patients and caregivers regarding treatment [47].

### Limitations

This combined review has several limitations, one of which is that the quality of the published literature varied and was hard to determine. Some studies included only small populations and no randomization, creating the potential for issues such as selection bias; therefore, conclusions surrounding some of the results presented should be interpreted with caution. Results were also often self-reported by the patients with no clinical validation of disease- and treatment-related factors, which may confound patient-reported outcomes and presents the possibility of confirmation or recall bias. Patients were generally not studied over long periods and, as MM changes over time, the results may not be generalizable to all patients in all settings.

Across the published literature, details regarding disparities in access to health care were lacking, which represents both an unmet need and a limitation. Several studies identified sex, age, ethnicity, and social factors as an influence on the health of patients with MM (ie, findings from studies in which patients of certain populations were overrepresented could be distorted). There were minimal data regarding single patients who live alone, for whom the burden of MM may be heightened. Furthermore, male patients are often overrepresented in MM studies, and wealthier, more educated, and proactive patients generally participate in studies investigating the patient voice. Health disparities could exist for women, patients who are less active in speaking on or addressing their condition, or those who are poorer and less educated [3,8,11,37,45].

Social media data exist outside the formal research context, are not generated to answer a specific research question, and are not regulated or peer-reviewed. There are also limitations in the search being restricted to English-language–only patient-reported information and, in terms of sampling in particular, self-selection bias; social media contributors may include a narrow band of patients who are willing to share their narratives on the web. Social media data are also reliant on patient self-identification and self-reporting, which may not be verifiable. Furthermore, social media data are limited by the availability of patient demographic and clinical characteristics. Age was not reported for all patients included in the study; therefore, it is unknown whether potential age-related aspects of patients’ MM and treatment experience may have influenced some of the key themes that emerged from the patient-reported information [28,48].

### Conclusions

This study provides valuable and up-to-date information on patient experiences and perspectives regarding the impact of MM and its treatment. Our findings are consistent with recent publications investigating patient perceptions of MM and its treatment [28,48]; patients are affected by side effects and uncertainties in treatment benefits, resulting in psychological and physical burden [48], yet they value some aspects such as the convenience of at-home versus hospital administration [28]. Patient-reported information shared on social networking platforms is unsolicited, publicly available data that can provide insight on the priorities of both patients and caregivers that may not always be captured by more traditional research methods such as interviews or surveys [49]. Furthermore, as patient-reported information is an existing source of data generated independently by individual users, it is not burdened by the limitations associated with interviewer bias or recall challenges [49]. Patient-reported information represents the unfiltered patient voice speaking or writing directly to a web-based audience about the topics of interest and importance to them. Therefore, it may provide a rich source of information about the patient experience that can complement traditional research methods.

The data from this combined review highlighted that the patient journey in MM is multifaceted; patients’ HRQOL is impeded not only by the symptoms and progression of the disease but also by treatment-related side effects, which can have a substantial and long-lasting impact on patients’ lives. The patient perspective on participation in treatment decisions is an important factor in the journey, and our research shows that, in published literature and on social media, patients appreciate involvement in deciding treatment options. Our review highlights the importance of further understanding patient perspectives on MM as this is an important area for improving the overall quality of care for patients.

## Appendix

Multimedia Appendix 1 Social media review key search terms.

Multimedia Appendix 2 Major themes and codes.

## Abbreviations

HRQOL: health-related quality of life
MM: multiple myeloma
RRMM: relapsed and refractory multiple myeloma

## Data Availability

The data sets generated and analyzed during this study are available from the corresponding author upon reasonable request.
