# Supplementary material for: Characterizing the Patient Journey in Multiple Myeloma: Qualitative Review
Source: JMIR Cancer. 2022 Sep 22;8(3):e39068. doi: 10.2196/39068 (PMC9539647; doi:10.2196/39068)
Supplement: Multimedia Appendix 1 [file cancer_v8i3e39068_app1.docx]

Supplementary Materials

Appendix Table 1. Social media review key search terms

| Concept | Key search terms |
| --- | --- |
|  |  |
| Patient experience | “patient,” “story,” “journey,” “narrative,” “experience,” “diary,” “blog,” “forum” |
| MM | “Multiple Myeloma,” “relapsed/refractory,” “MM” |
| Impact of MM/treatments | “impact,” “burden,” “effect,” “quality of life,” “family life,” “family activities,” “relationships” |
| Caregiver’s experience | “caregiver,” “care-partner,” “carer,” “family^a^,” “husband,” “wife” |
| MM treatments | “treatment,” “therapy,” “Chemotherapy,” “Radiation therapy,” “CAR-T*,” “stem cell transplant” |
| Treatment administration type | “oral,” “tablets,” “injection*,” “intra-venous,” “infusion” |
| Treatment satisfaction/preference | “satisfaction,” “fulfil^a^,” “happy,” “expect^a^,” “preference” |
| Treatment effectiveness | “effect^a^,” “improve symptoms,” “manage symptoms,” “delay progression” |
| COVID-19 pandemic | “COVID-19,” “Coronavirus,” “pandemic” |

^a^ Boolean operators will be used where supported by search function.

CAR-T = chimeric antigen receptor T-cell; MM = multiple myeloma.
