# Supplementary material for: Characterizing the Patient Journey in Multiple Myeloma: Qualitative Review
Source: JMIR Cancer. 2022 Sep 22;8(3):e39068. doi: 10.2196/39068 (PMC9539647; doi:10.2196/39068)
Supplement: Multimedia Appendix 2 [file cancer_v8i3e39068_app2.docx]

Supplementary Materials

Appendix Table 2. Major themes and codes

| Themes | Initial themes |
| --- | --- |
|  |  |
| Diagnosis and/or  disease history | Date of diagnosis, symptoms or signs leading up to diagnosis, duration of disease, disease progression |
| Symptoms |  |
| Impacts | **Physical functioning:** mobility, lifting, exercise, swallowing  **Relationships:** family, friends, partner, intimacy  **Social functioning:** socializing, leisure activities outside the home, social planning, life events (eg, weddings)  **Emotional impact:** depression, anxiety, frustration, fear  **Daily activities:** household chores, self-care (e.g., showering, bathing, dressing), running errands, childcare  **Sleep:** falling asleep, disturbed sleep, sleep quality  **Work:** Productivity, missing days of work, inability to work and/or financial impact |
| General treatment experience | Treatment history, current treatment, treatment types |
| Treatment administration | Treatment preferences, treatment effectiveness, treatment impacts, treatment side effects, treatment burden |
| Treatment outcomes | Treatment effectiveness, treatment satisfaction, treatment preferences, treatment expectations |
| Influences impacts | Side effects, treatment burden, travel burden |
| Impact on caregiver and/or family member | Relationships, social functioning, emotional impact, daily activities, sleep, work |
| COVID-19 pandemic | “COVID-19,” “Coronavirus,” “pandemic” |
